# Factors Affecting the Life Satisfaction of School-Aged Children with Vietnamese Immigrant Mothers in Korea

**DOI:** 10.3390/healthcare11172465

**Published:** 2023-09-04

**Authors:** Yoon-Hee Cho, Joohyun Lee

**Affiliations:** 1 Department of Nursing, College of Nursing, Dankook University, Cheonan 31116, Republic of Korea; choyoonhee@dankook.ac.kr; 2 Department of Nursing, College of Nursing, Eulji University, Seongnam 13135, Republic of Korea

**Keywords:** children, multicultural family, biopsychosocial model, MAPS 2 data

## Abstract

Low life satisfaction among multicultural children is an important issue related to children’s mental health in Korea. The purpose of this study was to identify factors influencing the life satisfaction of children whose Vietnamese mothers migrated to Korea for marriage. Data from the Multicultural Adolescents Panel Study (MAPS) conducted by the National Youth Policy Institute (NYPI) in 2020 were used. The participants were 586 elementary school students. The mean age was 11.01 years (SD 0.19), and 52.0% were girls. Based on Engel’s biopsychosocial (BPS) model, biological factors (gender, physical health, and body mass index), psychological factors (mental health, acculturative stress, self-esteem, and general stress), and sociocultural factors (family economic status, social support, and parenting style) were measured and analyzed by using hierarchical multiple regression. In the final model, self-esteem (β = 0.399, *p* < 0.001), parental social support (β = 0.184, *p* < 0.001), and neglectful parenting style (β = 0.085, *p* = 0.031) were positively related to children’s life satisfaction, whereas general stress (β = −0.213, *p* < 0.001) and acculturative stress (β = −0.104, *p* = 0.002) were negatively related. The overall explanatory power was 41.7%. In conclusion, it is necessary to develop a specialized program that focuses on strengthening the bonds between parents and their children to enhance family functioning for multicultural families.

## 1. Introduction

Korea’s traditional male preference has led to a gender imbalance in the marriage-age population over the last few decades, and rapid economic growth and industrialization have increased the numbers of unmarried people and of those who marry at an older age. Despite this social phenomenon, there are still many people who believe that, traditionally, adult men and women should marry and have children [1]. Reflecting this situation, international marriages have rapidly increased in South Korea, accounting for 10.3% of all marriages in 2019 [2]. In particular, international marriages between women from China or Southeast Asia and Korean men have increased [2]. The increase in international marriages has contributed to an increase in the number of children born to multicultural families, accounting for 6% of all births in 2020 [3].

The greatest proportion of foreigners married to Koreans were people coming from China, where there is a similar historical and cultural background and to which many Koreans migrated in the 19th and 20th centuries due to circumstances such as colonization and world wars [4]. Because of this historical background, foreigners from China tend to be more fluent in Korean than people from other countries are [5]. On the other hand, the highest percentage of international marriages from countries with fewer cultural similarities and different languages was from Vietnam, which accounted for 24.5% of all international marriages [4]. As Vietnamese women usually migrate for marriage after short relationships with Korean men, they have difficulty using the Korean language and have a high rate of conflicts related to cultural adjustment [6].

Marriages between speakers of different languages and members of different cultures are more likely to be unstable than those between speakers of the same language and members of the same culture [7]. In other words, difficulties in linguistic communication can lead to problems with self-expression and in understanding one’s spouse and Korean culture, and there are many problems in raising and educating children [7]. This also limits access to healthcare [8]. In 2021, the proportion of school-aged children aged 9–14 from multicultural families was 31.4%, an increase of about 6.5 percentage points from 2018 [3]. Furthermore, the proportion of school-aged children is expected to increase in the future, as the proportion of children under 9 years of age is around 50%. Therefore, attention to the physical and psychosocial health of these children is important for public health and the social integration of Korean society.

Immigration is associated with negative mental health outcomes, such as depressive symptoms and acculturative stress, regardless of age [9]. These mental health problems are associated with experiences of discrimination, acculturation, cultural differences, and loss of one’s community [10]. Due to developmental vulnerabilities, children from multicultural families may be more likely to experience negative mental health outcomes during childhood and adolescence. They are more likely to experience stress as a minority in Korean society and are at risk of lower life satisfaction [11]. Life satisfaction during childhood and adolescence is one of the most important indicators of quality of life and health [12]. This is because life satisfaction and happiness during this period can affect individuals throughout their lives [13]. However, life satisfaction among Koreans is not high [14], and it is believed that this is especially true for children and adolescents from multicultural families who are relatively vulnerable and are culturally different. According to previous studies, multicultural school-aged children in Korea had higher stress scores and lower self-esteem scores than those of children of Korean parents [15]. In addition, the school adjustment and life satisfaction of children of immigrant mothers in Korea differed according to the mother’s country of origin [16]. This may be because the cultural and linguistic experiences of school-aged children are related to their mental health and life satisfaction.

The biopsychosocial (BPS) model has gained wide acceptance in mental health, as well as in health education, health psychology, and public health [17]. Engel used the term BPS to describe the limitations of a strictly biological model of health and to emphasize a psychosocial approach [18]. The BPS model explains the development of disease through a complex interplay of biological (genetic, biochemical, etc.), psychological (mood, personality, behavior, etc.), and social (cultural, familial, socioeconomic, medical, etc.) factors [17,18,19]. In the sense that life experiences and current psychological and social situations are important for mental health [17], the BPS model is considered to be appropriate for explaining the life satisfaction of school-aged children from multicultural families in relation to their environment.

Therefore, the purpose of this study was to explore the factors that influence the life satisfaction of school-aged children in multicultural families with Vietnamese mothers based on the BPS model. The biological factors, psychological factors, and sociocultural factors proposed by Engel [19] were used to explain the life satisfaction of school-aged children. The factors identified in this study were the following: (1) biological factors—gender, physical health, and body mass index (BMI); (2) psychological factors—mental health, acculturative stress, self-esteem, and general stress; (3) sociocultural factors—family economic status, social support (parents, teachers, and friends), and parenting style (supervising vs. neglectful). In this study, we assumed that psychological factors and sociocultural factors would have a significant impact on the life satisfaction of multicultural children based on the BPS model.

## 2. Materials and Methods

### 2.1. Study Design

This was a cross-sectional descriptive study that analyzed secondary data to identify factors that influenced the life satisfaction of school-aged children of Vietnamese mothers who migrated to Korea for marriage.

### 2.2. Participants

The participants in this study were 586 fifth-grade elementary school students with Vietnamese mothers and Korean fathers; Vietnamese women make up the largest number of marital migrants in Korea after Chinese women. We used data from the second wave of the Multicultural Adolescents Panel Study (MAPS) conducted by the National Youth Policy Institute (NYPI) of Korea in 2020 [20]. The mean age of the participants was 11.01 years (SD 0.19), ranging from 10 to 13 years, and 52.0% of the participants were girls.

### 2.3. Data Collection and Research Ethics

This study used data from the second year of the MAPS survey in South Korea, which was conducted in 2020. The second wave of the MAPS survey included 2271 children of international marriages, mid-career immigrants, and foreigners. Data from 586 children with Korean fathers and Vietnamese mothers who immigrated to Korea for marriage were extracted for this study. The data were used after the NYPI reviewed the researcher’s study plan and gave permission to use the data. All data were used after a confidentiality agreement and were kept inaccessible to all except the researchers. In addition, all data were de-identified in accordance with the Korean Personal Information Protection Act and Statistics Act. Data use and analysis followed the standards of the user guide provided by the NYPI [21].

### 2.4. Tools

#### 2.4.1. Life Satisfaction

Life satisfaction is defined as an overall assessment of the quality of life according to an individual’s judgment and standards, and it is a subjective feeling of satisfaction that life is enjoyable, there are few worries, and life is good [22]. In this study, life satisfaction was measured by using an instrument developed by Kim et al. [23]. The scale consists of 3 items: ‘I think my life is happy’, ‘I enjoy living’, and ‘I don’t have many worries’, on a 4-point Likert scale. A higher total score indicates a higher level of life satisfaction. In a previous study [23], Cronbach’s α was 0.75, and in this study, Cronbach’s α was 0.75.

#### 2.4.2. Biological Factors

The biological factors included gender, physical health, and BMI. Physical health was measured by using a 4-point Likert scale of the child’s perceived overall physical health. BMI was calculated from the child’s self-reported height and weight.

#### 2.4.3. Psychological Factors

Psychological factors included mental health, self-esteem, and general stress. Mental health was measured by using a 4-point Likert scale to assess the child’s perceived overall mental health. Self-esteem and general stress were measured by using scales from the 2018 National Youth Survey in South Korea [24]. Self-esteem was measured by using a 4-point Likert scale with 3 items on whether the child felt that he or she was a valuable person. Cronbach’s α in this study was 0.80. General stress was measured by using a 4-point Likert scale with 2 items asking about stress in everyday life and in the last 2 weeks, with a Cronbach’s α of 0.67.

#### 2.4.4. Sociocultural Factors

The sociocultural factors identified in this study were family economic status, social support, parenting style, and acculturative stress. Family economic status was measured according to the child’s subjective economic level on a 5-point Likert scale. Children’s social support consisted of support from parents, teachers, and friends. The measurement consisted of 6 items for parental support, 3 items for teacher support, and 3 items for friend support, with higher total scores indicating higher social support. Cronbach’s α for the measurement was 0.88 for parental support, 0.83 for teacher support, and 0.86 for friend support. Parenting style was assessed by categorizing parents’ interests and attitudes towards their children as either neglectful (uninvolved) or providing appropriate supervision, such as by caring about their children and monitoring their children’s lives [25]. Neglectful parenting attitudes were measured by using an instrument based on part of the child maltreatment questionnaire developed by Huh [25] and modified by the NYPI [26]. Neglectful parenting attitudes were a single factor that included parents’ interest in their children, emotional support, meeting needs, medical support, interest in school, and support in life. It consisted of a total of 7 items, with representative items including ‘My parents (guardians) are not there when I need them’ and ‘My parents (guardians) don’t care about me and never praise or punish me’. The level of response to each item was based on a 4-point Likert scale ranging from ‘not at all true’ to ‘very true’. Two of the seven items were reverse questions about neglectful parenting, so the items were reverse-scored and used. The higher the sum of the values of each item, the higher the level of neglectful parenting attitude in the parenting behavior. Cronbach’s α was 0.76. The supervising parenting attitude consisted of 3 items: ‘My parents know where I go after school’, ‘My parents know how I spend my time’, and ‘My parents know when to expect me home when I go out’. The greater the sum of the scores of each item, the higher the level of supervisory parenting in parenting attitudes. Cronbach’s α was 0.77. Acculturative stress was measured by using an instrument developed by Hong [27]. The scale consisted of 9 items on a 4-point Likert scale. Representative items included ‘I feel stressed when others make jokes about the culture of my foreign parents’ country’, ‘I feel stressed because I don’t speak Korean well’, and ‘I feel stressed when I have to act like a Korean in front of others’. A higher total score indicated a higher level of acculturative stress. Cronbach’s α was 0.71 in a previous study [27], and in this study, Cronbach’s α was 0.84.

### 2.5. Statistical Analysis

The data collected were analyzed by using IBM SPSS, version 27.0 (IBM, Armonk, NY, USA). Participants’ characteristics, life satisfaction, and independent variables were analyzed by using descriptive statistics with numbers and percentages or means and standard deviations. Differences in life satisfaction and independent variables by gender were analyzed with an independent t-test. Correlations between participants’ life satisfaction and biological, psychological, and sociocultural factors were analyzed by using Pearson’s correlation coefficients. Finally, factors influencing participants’ life satisfaction based on Engel’s BPS model were analyzed by using hierarchical multiple regression analysis. Biological factors were analyzed in model 1, biological factors and psychological factors were analyzed in model 2, and biological factors, psychological factors, and sociocultural factors were included in model 3. Tolerance (>0.1), VIF (<10), and Durbin–Watson values (a value of 2 or close to 2 indicates that there is no autocorrelation) were used to check whether the assumptions of the multiple regression analysis were met, and the level of statistical significance was less than 0.05.

## 3. Results

### 3.1. Life Satisfaction and Biological, Psychological, and Sociocultural Factors of the Participants

The life satisfaction of children of Vietnamese mothers and Korean fathers was 3.24 (SD 0.53), with no difference according to gender. Among the biological factors, the BMI was 20.30 (SD 3.82) on average, with boys (21.10) reporting higher values than girls (19.57). Physical health was 3.45 (SD 0.60), with girls (3.50) reporting higher values than boys (3.40). Among the psychological factors, mental health was 3.45 (SD 0.57), self-esteem was 3.23 (SD 0.52), and general stress was 3.53 (SD 1.45). None of the variables of the psychological factors differed by gender. Among the sociocultural factors, the children’s perceived family economic level was 2.77 (SD 0.65), which was around the median. Social support from parents was 3.19 (SD 0.53), with no difference according to gender. However, social support from teachers was 3.73 (SD 0.71), and social support from friends was 3.79 (SD 0.20), with girls reporting higher social support than boys. Parental attitudes were 3.17 (SD 0.62) for the supervising style and 1.97 (SD 0.46) for the neglectful style. Children’s perceived acculturative stress was 1.23 (SD 0.33) (Table 1).

### 3.2. Correlations between Life Satisfaction and Biological, Psychological, and Sociocultural Factors

Among the biological factors, physical health was positively correlated with children’s life satisfaction (r = 0.325, *p* < 0.001), while BMI was not correlated with it. Among the psychological factors, general stress was negatively correlated with life satisfaction (r = −0.339, *p* < 0.001), while mental health (r = 0.400, *p* < 0.001) and self-esteem (r = 0.577, *p* < 0.001) were positively correlated with it. Among the sociocultural factors, perceived family economic level (r = 0.100, *p* = 0.015), social support from parents (r = 0.401, *p* < 0.001), teachers (r = 0.276, *p* < 0.001), and friends (r = 0.304, *p* < 0.001), and supervising parenting style (r = 0.279, *p* < 0.001) were positively correlated with the life satisfaction of children. On the other hand, neglectful parenting style (r = −0.232, *p* < 0.001) and acculturative stress (r = −0.261, *p* < 0.001) were negatively correlated with life satisfaction) (Table 2).

### 3.3. Factors Influencing Life Satisfaction of School-Aged Children of Vietnamese Immigrant Mothers

Table 3 shows the factors influencing the life satisfaction of school-aged children with immigrant Vietnamese mothers. Before conducting the hierarchical multiple regression to determine the factors influencing participants’ life satisfaction, we checked the assumptions about the independent variables in the regression analysis. The Durbin–Watson value was 1.857, indicating that there was no autocorrelation. In addition, the tolerance values ranged from 0.50 to 0.98, which were above 0.10, and the variance inflation factor values ranged from 1.025 to 2.021, which were below 10, confirming that there was no multicollinearity. Based on Engel’s BPS model [19], this study analyzed biological factors in model 1, biological factors and psychological factors in model 2, and biological factors, psychological factors, and sociocultural factors in model 3. In model 1, among the biological factors, physical health had a significant effect on participants’ life satisfaction (β = 0.328, *p* < 0.001), with an explanatory power of 10.3% for total life satisfaction. In model 2, biological factors had no effect on life satisfaction, and among the psychological factors, mental health (β = 0.115, *p* = 0.011), self-esteem (β = 0.447, *p* < 0.001), and general stress (β = −0.228, *p* < 0.001) had significant effects, with an overall explanatory power of 39.3%. Finally, in model 3, among the psychological factors, self-esteem (β = 0.399, *p* < 0.001) and general stress (β = −0.213, *p* < 0.001) influenced life satisfaction, and among the sociocultural factors, parental social support (β = 0.184, *p* < 0.001), neglectful parenting style (β = 0.085, *p* = 0.031), and acculturative stress (β = −0.104, *p* = 0.002) influenced life satisfaction. The total explanatory power for life satisfaction in model 3 was 41.7%.

## 4. Discussion

This study was conducted to explore various factors affecting the life satisfaction of school-aged children with Vietnamese mothers based on the BPS model. The levels of life satisfaction and biological, psychological, and sociocultural factors of the participants were measured, and the effect of each factor on life satisfaction was analyzed by using hierarchical regression analysis. First, the average life satisfaction score for the children of Vietnamese mothers was 3.24. In another study that used the same life satisfaction scale [28], the average life satisfaction score of all multicultural adolescents, including children of Vietnamese mothers, was 3.33. In another study of a panel of multicultural adolescents using the same scale [15], the average life satisfaction score was 3.35. These findings were consistent with those of a previous study that found that the life satisfaction of children of immigrant mothers differed according to the mother’s country of origin [16]. Parents in multicultural families—especially women who migrate for marriage—may experience acculturation stress and pass it on to their children [29]. The children of Vietnamese mothers might have more difficulties in adapting to Korean society due to their mothers’ limited language skills and cultural heterogeneity than the children of Chinese mothers, who are more accustomed to the Korean language and culture due to their historical background. Therefore, it is suggested that the overall level of acculturation should be assessed before designing programs to increase cultural competence in multicultural families, as there might be a difference depending on the mother’s country of origin.

In this study, the life satisfaction scores of 3.24 for boys and 3.23 for girls showed that there were no gender differences, which was similar to the findings of a previous study [30]. In this other study, there were no gender differences in life satisfaction during the elementary school years. The age range of the participants in this study was 10 to 13 years old, which corresponded to grades four to six of elementary school. However, in another study [30], in the middle school period, boys scored higher than girls in the first year. In another study comparing the life satisfaction of adolescents, it was found that life satisfaction scores were significantly different according to gender in multicultural and non-multicultural adolescents [31]. Therefore, although gender differences in life satisfaction were not particularly pronounced in the elementary school years in this study, future studies would be needed to explore gender differences in later age groups—especially adolescents.

Parental support did not significantly differ according to gender in this study, but social support from teachers and friends was reported to be significantly higher for girls than for boys. These findings were consistent across the multicultural sample, with girls reporting significantly more support from teachers and friends than boys and with boys reporting higher rates of friendship difficulties (13.5% vs. 10.9%) [32]. Park’s study explored the relationship between social support and life satisfaction and reported that support from friends had a positive effect on life satisfaction for girls, whereas neither support from teachers nor support from friends had a significant effect on life satisfaction for boys. Although this study did not examine gender differences in the relationship between social support and life satisfaction, gender was found to be a non-significant factor in life satisfaction in the final regression analysis. This finding may be related to the fact that there were no significant gender differences in life satisfaction during the elementary school years [30].

In this study, overall stress, neglectful parenting style, and acculturation stress were negatively associated with life satisfaction, while mental health, self-esteem, family economic level, social support from parents, teachers, and friends, and supervisory parenting style were positively associated with life satisfaction. These findings are similar to those reported in the 2012 Korean Children and Youth Panel Survey, where the environmental characteristic variables of parental closeness, teacher closeness, friendship, and academic adjustment were found to have a positive impact on children’s life satisfaction [33]. The variables that had the greatest impact were, in order, support from friends, adjustment to learning, support from parents, and support from teachers.

Based on the BPS model, which holds that life experiences are influenced by a complex interplay of biological, psychological, and social factors [17,18], biological factors were first analyzed in model 1. Physical health had a significant effect on life satisfaction, with an explanatory power of 10.3%. However, when biological factors and psychological factors were analyzed in model 2, physical health had no significant effects on life satisfaction. Instead, all psychological factors, including mental health, self-esteem, and general stress, had a significant effect on life satisfaction, with an increase in explanatory power of up to 39.3%. Finally, when biological factors, psychological factors, and sociocultural factors were included in model 3, the variables that significantly influenced the life satisfaction of children with Vietnamese mothers were self-esteem and general stress among the psychosocial factors, as well as parental support, neglectful parenting attitudes, and acculturation stress among the sociocultural factors, with an increase in the explanatory power of up to 41.7%. These findings are consistent with the assumption of the BPS model that, in addition to physiological factors, psychological and sociocultural factors also play an important role in mental health [19].

The factors that significantly increased life satisfaction were self-esteem, parental support, and neglectful parenting attitudes, while the factors that significantly decreased life satisfaction were general stress and acculturation stress. These results are consistent with the findings of a previous study [34]. They reported that parental support, mental health satisfaction, and self-satisfaction had positive effects on life satisfaction among adolescents from multicultural families. Self-satisfaction and social competence among multicultural adolescents were reported to have a significant static relationship and to mediate the relationship between attitudes of cultural acceptance and life satisfaction [32]. Parental support and positive parenting attitudes were reported to be strongly associated with the development of children’s self-esteem [35], to positively influence school adjustment, and to reduce the risk of school dropout by mediating self-esteem [36].

In this study, the level of neglectful parenting attitudes was 1.97, and the final regression analysis showed that it had a significant positive effect on the life satisfaction of children of Vietnamese mothers. Parental attitudes have many effects on children’s emotional development, and negative parenting attitudes, such as inconsistency and over-interference, have been reported to be associated with negative emotions, such as depression, in children, which, in turn, leads to poorer school adjustment [37]. Furthermore, although parental support was a major social relationship factor for both boys and girls, parental supervision had a significant static effect on life satisfaction only for boys [34]. Interestingly, their study found that the level of parental supervision had little direct effect on life satisfaction or mediated the effect of acculturative stress on life satisfaction for girls, but for boys, higher levels of parental supervision had a significant static effect on life satisfaction and slowed a decline in life satisfaction caused by acculturative stress. The positive effect of neglectful parenting attitudes on the life satisfaction of children with Vietnamese mothers in this study, although the effect size is small, could be attributed to these gender differences in parental supervision and life satisfaction. Therefore, when developing parenting support strategies to increase children’s life satisfaction in multicultural families, it is necessary to develop gender-specific content so that it can be selectively applied.

The study found that acculturative stress had a significant negative impact on participants’ life satisfaction, with a level of 1.23. In a study of adolescents from multicultural families, maternal acculturation stress negatively affected children’s emotions [7] and mediated depression, which, in turn, negatively affected children’s school adjustment [30]. Multicultural children experienced acculturation stress due to social prejudice, discrimination, and identity concerns, which led to school maladjustment and decreased life satisfaction [33]. Acculturation stress in multicultural children was also associated with the development of emotional problems such as depression or anxiety [11,38] and low self-esteem [35]. It is, therefore, necessary to continue to implement cultural diversity education programs in schools in order to increase multicultural acceptance among school members, in addition to developing and implementing various local programs to increase multicultural acceptance at the community level.

This study has some limitations, as it is a secondary analysis study using MAPS data. Firstly, this study conducted a cross-sectional analysis using only the second-year data from the second-term MAPS survey. Therefore, time-series changes and causal relationships were not considered. Secondly, we were unable to examine all factors (e.g., parental and family characteristics, parent–child attachment, and school life) reported in previous studies due to limitations in the secondary analysis. Thirdly, even though the physical and mental health variables tested in this study included various perspectives, they may not have been accurately identified as components of health status because they were measured with a single question.

## 5. Conclusions

In this study, the life satisfaction of school-aged children of Vietnamese mothers was found to be influenced by children’s self-esteem, general and acculturative stress, parental social support, and neglectful parenting attitudes based on the BPS model. Therefore, in order to increase the self-esteem of multicultural children and improve parental social support, it would be helpful to develop a specialized program to enhance family functioning for multicultural families while focusing on strengthening the bonds between parents and their children. Interestingly, this study found that neglectful parental attitudes had a positive effect on children’s life satisfaction. These findings suggest that parenting attitudes may have different effects on the life satisfaction of multicultural children depending on their gender, and further research on the life satisfaction of multicultural children according to parenting attitudes by age and gender is needed to clarify this. Finally, at the level of the sociocultural system in Korea, continued efforts are needed to implement educational programs in schools and local communities to increase multicultural acceptance among all members of society.

## Figures and Tables

**Table 1:** Biological, psychological, and sociocultural levels and life satisfaction of participants according to gender.

Variables	Total	Boys	Girls	t (*p*)
Life satisfaction	3.24 ± 0.53	3.24 ± 0.51	3.23 ± 0.55	0.181 (0.857)
BMI	20.30 ± 3.82	21.10 ± 4.18	19.57 ± 3.30	4.913 (<0.001)
Physical health	3.45 ± 0.60	3.40 ± 0.60	3.50 ± 0.60	−2.014 (0.044)
Mental health	3.45 ± 0.57	3.41 ± 0.55	3.49 ± 0.59	−1.750 (0.081)
Self-esteem	3.23 ± 0.52	3.22 ± 0.52	3.23 ± 0.52	−0.338 (0.736)
General stress	3.53 ± 1.45	3.56 ± 1.42	3.50 ± 1.47	0.478 (0.633)
Perceived family economic level	2.77 ± 0.65	2.77 ± 0.67	2.77 ± 0.63	−0.034 (0.973)
Social support from parents	3.19 ± 0.53	3.16 ± 0.53	3.21 ± 0.52	−1.192 (0.234)
Social support from teachers	3.73 ± 0.71	3.64 ± 0.66	3.81 ± 0.74	−2.910 (0.004)
Social support from friends	3.79 ± 0.72	3.66 ± 0.76	3.90 ± 0.66	−4.054 (<0.001)
Parenting style (supervising)	3.17 ± 0.62	3.13 ± 0.61	3.21 ± 0.64	−1.692 (0.091)
Parenting style (neglectful)	1.97 ± 0.46	2.01 ± 0.48	1.94 ± 0.45	1.911 (0.056)
Acculturative stress	1.23 ± 0.33	1.25 ± 0.36	1.21 ± 0.31	1.289 (0.198)

**Table 2:** Correlations of biological, psychological, and sociocultural factors with life satisfaction.

Variables	Life Satisfaction
	r (*p*)
Biological factors	
BMI	0.029 (0.478)
Physical health	0.325 (<0.001)
Psychological factors	
Mental health	0.400 (<0.001)
Self-esteem	0.577 (<0.001)
General stress	−0.339 (<0.001)
Sociocultural factor	
Perceived family economic level	0.100 (0.015)
Social support (parents)	0.401 (<0.001)
Social support (teachers)	0.276 (<0.001)
Social support (friends)	0.304 (<0.001)
Parenting style (supervising)	0.279 (<0.001)
Parenting style (neglectful)	−0.232 (<0.001)
Acculturative stress	−0.261 (<0.001)

**Table 3:** Factors influencing life satisfaction.

Variables	Model 1		Model 2		Model 3	
	β	t (*p*)	β	t (*p*)	β	t (*p*)
(Constant)		11.802 (<0.001)		7.241 (<0.001)		4.007 (<0.001)
Biological factors						
Sex (Ref: boys)	−0.028	−0.691 (0.490)	−0.029	−0.753 (0.452)	−0.029	−0.887 (0.376)
BMI	0.037	−0.928 (0.354)	0.031	1.334 (0.183)	0.054	1.672 (0.095)
Physical health	0.328	8.350 (<0.001)	0.082	1.949 (0.052)	0.061	1.446 (0.149)
Psychological factors						
Mental health			0.115	2.546 (0.011)	0.083	1.870 (0.062)
Self-esteem			0.447	12.564 (<0.001)	0.399	10.582 (<0.001)
General stress			−0.228	−6.838 (<0.001)	−0.213	−6.324 (<0.001)
Sociocultural factor						
Perceived family economic level					−0.014	−0.432 (0.666)
Social support (parents)					0.184	4.086 (<0.001)
Social support (teachers)					−0.022	−0.551 (0.582)
Social support (friends)					0.041	1.026 (0.305)
Parenting style (supervising)					−0.021	−0.523 (0.601)
Parenting style (neglectful)					0.084	2.162 (0.031)
Acculturative stress					−0.104	−3.065 (0.002)
Adjusted R^2^	0.103		0.393		0.417	
F	23.432		64.147		33.120	
*p*-value	<0.001		<0.001		<0.001	

## Data Availability

Data sharing is not applicable.
